# Optical coherence tomography homography for detection of retinal displacement: a validation study

**DOI:** 10.1007/s00417-024-06555-1

**Published:** 2024-07-10

**Authors:** Koby Brosh, Eduardo Roditi, Aditya Bansal, Isabela Martins Melo, Michael J Potter, Rajeev H. Muni

**Affiliations:** 1 Department of Ophthalmology, Shaare Zedek Medical Center, Faculty of medicine, Hebrew University of Jerusalem, Jerusalem, 9103102 Israel; 2 Universidad Anáhuac México Norte, México City, CDMX México; 3 Department of Ophthalmology, St. Michael’s Hospital, Unity Health Toronto, Toronto, ON Canada; 4 Department of Ophthalmology and Vision Sciences, University of Toronto, Toronto, ON Canada; 5 Department of Ophthalmology and Visual Sciences, University of British Columbia, Vancouver, Canada; 6 Department of Ophthalmology and Visual Science, University of Kentucky, Lexington, USA; 7 Retinal Consultant, Active Vision Center, México City, México

**Keywords:** Retinal displacement, Retinal detachment, Ocular coherence tomography (OCT), Overlay, Infrared imaging

## Abstract

**Purpose:**

Retinal displacement following rhegmatogenous retinal detachment (RRD) has been associated with inferior functional outcomes. Recent evidence using an overlay technique suggests that fundus-autofluorescence underestimates post-RRD repair retinal displacement. This study aims to validate the overlay technique in normal eyes and to determine its sensitivity and specificity at detecting retinal displacement.

**Methods:**

We conducted a retrospective case series involving 66 normal eyes, each with at least two separate infrared (IR) images at different time points. Overlay of the two images was based on manual marking of choroidal and optic nerve head (ONH) landmarks. For each set of two IR images, computer code for homography generated two outputs, flipping view video and an overlay picture. First, validation of choroidal/ONH alignment was performed using the flipping view video to ensure accurate manual markings. Then, two different masked graders (AB + IM) evaluated the overlays for presence of retinal displacement. 16 control eyes following RRD repair with detected retinal displacement on FAF imaging assessed sensitivity and specificity of the technique.

**Results:**

94% of overlays were found to be well aligned (62/66). 11 cases exhibited errors on flipping view analysis (choroidal/ONH misalignment). Those 11 cases had a significantly higher rate of retinal displacement (false positives) compared to cases without errors (8/11,72% Vs 54/55,98%,*P* = 0.001). Sensitivity and specificity of the overlay technique for detecting retinal displacement considering only adequate flipping view cases (*n* = 55) were calculated as 100% and 98%, respectively.

**Conclusions:**

IR overlay emerges as a reliable and valid method for detecting retinal displacement, exhibiting excellent sensitivity and specificity.

**Supplementary Information:**

The online version contains supplementary material available at 10.1007/s00417-024-06555-1.

## Key message

***What is known***

•Fundus autofluorescence has a low sensitivity for detection of displacement following RRD repair.
•Overlay of two infrared images (homography) is a promising technique that may be used in many retinal pathologies to reveal retinal misalignment.

***What is new***

•Infrared images at different timepoints can be aligned using homography in normal eyes with excellent sensitivity and specificity for detecting retinal displacement.
•To reduce false positives, homography should be accompanied by the flipping view technique to verify proper alignment of the choroid and optic nerve.

**Supplementary Information:**

The online version contains supplementary material available at 10.1007/s00417-024-06555-1.

## Introduction

Despite the high success rate of primary rhegmatogenous retinal detachment (RRD) repair, recent studies have highlighted adverse events regarding postoperative anatomic integrity and functional outcomes [1–4]. Recent advances in imaging technology have enabled the detection of subtle abnormalities in the post-reattachment position of the retina overlying the retinal pigment epithelium (RPE) [5, 6]. Prior studies have indicated a significant correlation between post-RRD retinal displacement and functional outcomes, highlighting the potential to reduce postoperative visual distortion by minimizing retinal displacement [7–11]. The detection of retinal displacement typically relies on fundus autofluorescence (FAF) imaging, through the identification of retinal vessels printings (RVPs), which represent the previous location of retinal blood vessels before the RRD occurred [5, 12]. Postoperative retinal displacement has been observed across different surgical techniques, with pars plana vitrectomy (PPV) often associated with higher rates (35–70%) [3] compared to pneumatic retinopexy (PnR) (7–15%) [7, 8] and scleral buckle (SB) (16.6%) procedures [13]. Recently, concerns have been raised regarding the sensitivity of FAF imaging in detecting retinal displacement, particularly in cases of subtle RVPs and sparse RPE mosaic, necessitating the exploration of alternative imaging modalities [6, 14].

Tung et al. introduced a novel overlay technique to measure foveal displacement in eyes undergoing epiretinal membrane surgery [15]. They utilized a computer program to overlay infrared images before and after surgery based on choroidal and optic nerve landmarks. Their technique used an overlay, based on deep choroidal landmarks to assess displacement of the retina, but they did not validate their technique in normal eyes. Previously, we used a similar method in eyes with available IR images prior to the occurrence of the rhegmatogenous retinal detachment (RRD) and overlaid post-RRD repair images to evaluate the sensitivity and specificity of fundus autofluorescence imaging (FAF) in detecting retinal displacement [14]. We demonstrated that FAF imaging diagnosed retinal displacement with low sensitivity and a high false negative rate. Furthermore, we found that the displacement rate (100%, 16/16) after pars plana vitrectomy (PPV) for RRD far exceeded what has been previously reported in the medical literature (35–70%) [3]. Clearly, there is a need to validate a technique for evaluating retinal displacement in normal and post-surgical eyes, and the homography procedure seems to offer obvious benefits over FAF alone [14]. To the best of our knowledge this is the first study that assesses the homography technique on two IR images longitudinally over time in normal patients.

## Methods

A retrospective case series of normal eyes was performed at the Shaare Zedek Medical Center (Jerusalem, Israel), a large tertiary care hospital. This study was approved by the Research Ethics Board and adhered to the Declaration of Helsinki [SZMC-0098-22].

Patients older than 18 years of age who had at least two separate macular SD-OCT scans co-registered with an IR image (Heidelberg, Germany, OCT Spectralis) at different timepoints were included. The OCT acquisition protocol consisted of macular cube composed of 49 horizontal 5-mm raster scans co-registered with an IR image. The two separate IR images were taken at least two weeks apart. It was required that all images did not demonstrate any macular pathology. Both eyes were included if they met the inclusion criteria. Exclusion criteria for the cohort of normal eyes included previous vitrectomy or scleral buckle, media opacity, pre-existing macular or retinal pathology such as epiretinal membrane or macular hole, and poor-quality images. Axial length measurements were available in 55 eyes.

The primary outcome was the alignment rate of normal IR images. Secondary outcomes included calculated sensitivity, specificity, positive and negative predictive value of the overlays, and the agreement rate between graders.

**Fig. 1:**
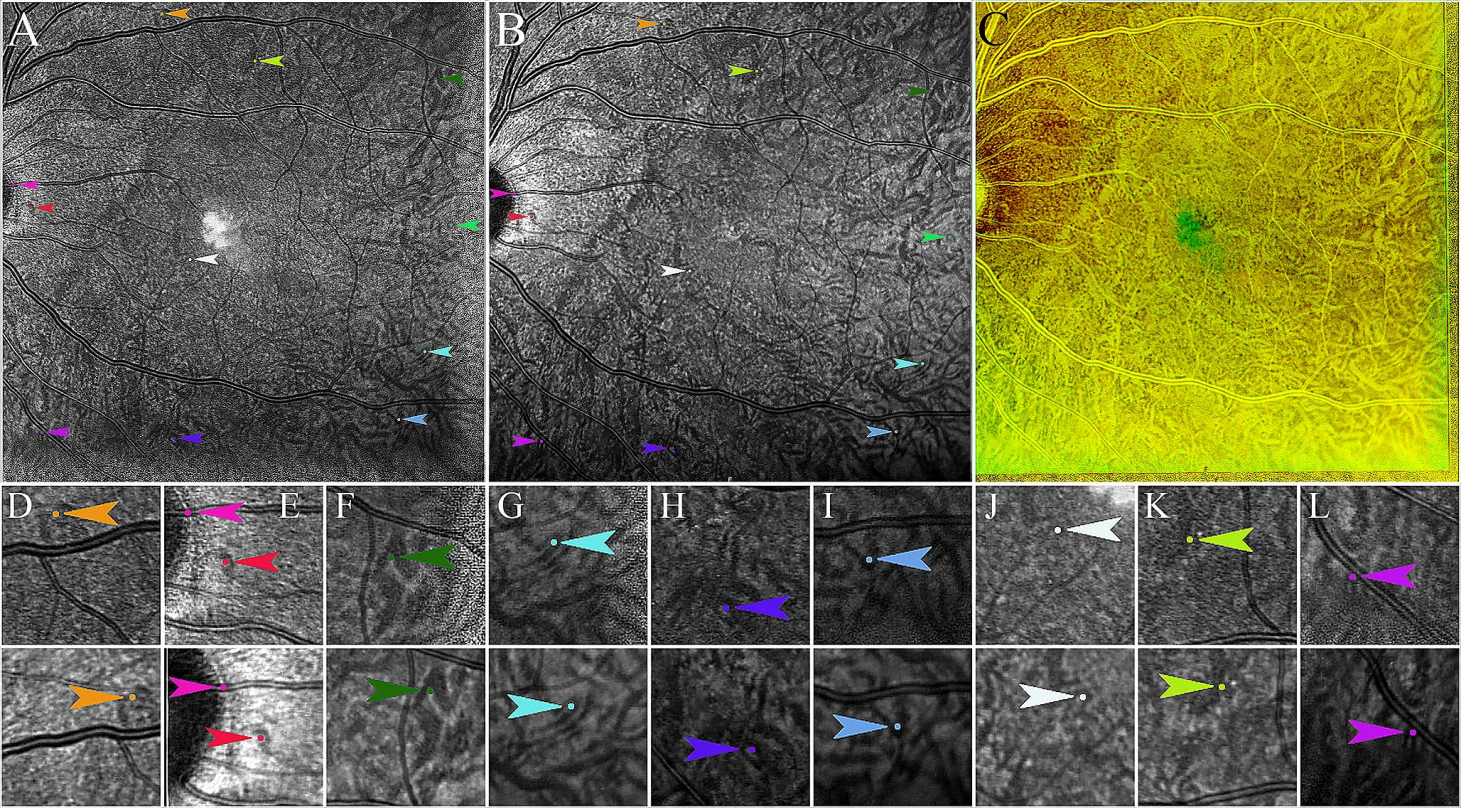
The homography technique. Two Infrared images of a healthy subject (**a** and **b**) with corresponding ONH and choroidal landmarks using matching colors to guide the overlay homography. Pairs of colored arrowheads point to matching landmarks (**c**) The final homography image demonstrates well-aligned retinal vessels. (**d**-**l**) Zoomed-in crops of IR images sections (**A** and **B**) are provided to visualize magnified corresponding landmarks on the ONH, RPE and choroid with upper and lower rows demonstrating matching points. For example, the yellow arrowheads indicate matching choroidal vessel bifurcations (K)

*Homography* was defined as the technique to generate the overlay of two separate IR images using a computer program (Fig. 1) [14]. The overlays were conducted based on deep (choroidal) landmarks which enabled assessment of retinal vessels alignment. At least four corresponding and matching deep landmarks were manually selected on the two IR images. We applied at least one landmark on the optic nerve heads (ONH) and at least four additional landmarks on choroidal vessels and/or RPE, with at least one landmark in each of the four different quadrants. Additional choroidal or RPE landmarks were used when available. Only obvious corresponding landmarks that matched between the two IR images in a given patient were chosen such, as choroidal vessel bifurcations. Homography was performed with a self-designed software program using Python coding to obtain the best overlay of the two IR images, based on the landmarks. Two different color channels (red and green) were used to readily distinguish the separate images, and their alignments.

In each set of two marked corresponding images, the computer software generated a “flipping view” whereby the readers could rapidly switch between the images and get a visual impression of the alignment between them (supplementary material) in addition to the overlay image.

The image analysis consisted of two steps: first step (flipping view video) to validate that the deep layers (choroid/RPE/ONH) are well-aligned and no flaws were generated in the overlay technique by the manual markings. The second step was to qualitatively assess the overlays for retinal vessels alignment, e.g. superior or inferior displacement, and the amount of displacement in terms of retinal vessel diameter.

The first step of image analysis was done by two independent masked graders (KB and ED) who assessed the choroidal and ONH alignment in the flipping view video. During this step, the graders assessed only the alignment of the deep layers of the choroid and ONH. In cases of sectoral misalignment, the landmarks in that sector were revised, and a new overlay and flipping view were generated.

In the second step, two different masked graders (AB and IM) independently assessed the color overlays for the presence or absence of retinal blood vessel misalignment including the extent and obviousness of the displacement. The graders were masked as to the displacement status of the patients, and whether images were from study or control cases. The same masked graders read a control group of (*n* = 16) overlays from patients from a previous study [14] known to have retinal displacement on FAF imaging after RRD repair. The control group was read together with the study group, so that the graders would not know whether any retinal pathology was expected. All the controls had two available IR images, one prior to the occurrence of RRD and the second following RRD repair. When agreement between the graders was not achieved, a third senior masked grader (KB) made the final assessment of retinal alignment.

The sensitivity and specificity of the technique were calculated using a reference standard (ground truth) established by a cohort of 16 control eyes with known retinal displacement after RRD repair.

The analysis of all overlays included determining the direction of the displacement (superior or inferior); quantifying displacement at each major arcade, typically the major retinal vein (greater or less than a vessel diameter); assessing the number of displaced vessels within the macula area between the two arcades and evaluating the obviousness of retinal displacement (faint, moderate or obvious). Graders assessed the obviousness of displacement based on the ease of detection.

Statistical analysis: The sample size of the study was calculated to detect a deviation with an effect size of 0.45, alpha of 0.05 and power of 0.95 (*n* = 66). All data was checked for normality (Shapiro-Wilk test). A-parametric tests were used on data with non-normal distribution. Descriptive statistics used were mean with standard deviation for normally distributed data, and median with interquartile range for non-normally distributed data. All inferential statistical analyses were conducted using two-sided P values. Interobserver agreement was assessed with Cohen’s kappa coefficient. Univariable analysis assessed the possible correlation between different variables (e.g. axial length, age, interval between the two images acquisition and the number of landmarks) and the primary outcome. All statistics were performed using statistical software IBM SPSS version 26 (Armonk, NY: IBM Corp., 2019).

## Results

66 normal eyes of 55 patients met the inclusion criteria. Patient characteristics were as follows: ages ranged from 44 to 91years (median 67 (62-74.5)), laterality was divided equally between the right and left eyes at 33 each, 23 patients were females and 32 were males, axial length measurements were available in 55 eyes and ranged from 21.88 to 29.36 mm (median 23.84 (22.85–24.88)). Two sets of IR images were available for in study cases of 10 glaucomatous eyes, 34 routine studies prior to phacoemulsification surgery, and 22 had a previous RRD in the fellow eye. Two sets of IR images were also available in control cases before and after RD repair in our previous study [14]. The interval between the two sets of the study IR images ranged from 2 to 227 weeks (median 20.5 (9.4–52.5)). Between 5 and 12 corresponding landmarks were marked on both sets of study IR images (median 9 (7–10)).

Preliminary image analysis included validation of stationary landmarks using the flipping view technique. The results of this step revealed 45 cases with stationary ONH/choroid landmarks and 11 cases with ONH/choroid misalignment. 10 eyes were found with sectoral choroidal misalignment in the flipping view, so revision of the landmarks in that sector was performed until the appeared stationary (Video in Supplementary Material). A total of 55 (45 + 10) eyes were stationary on the flipping view analysis. The remaining 11 of 66 eyes were found to have flipping view errors.

Further image analysis included grading of the overlays for retinal alignment. Overall, 94% (62/66) of the overlays were found to be aligned. There was a significant difference in overlay alignment between cases with or without errors in the flipping view analysis. 98% (54/55) of overlays with an adequate flipping view were found to be aligned compared to 73% (8/11) of overlays which had errors on the corresponding flipping view (*p* = 0.001). All true positive controls with known retinal displacement on FAF after PPV for RRD repair were found to have retinal misalignment on homography testing by masked graders.

The agreement rate between the two independent graders was 88% and 97% in the flipping mode analysis and the overlay analysis respectively (Cohen kappa 0.64, 0.94).

The sensitivity, specificity, positive and negative predictive value of the homography technique were calculated on the entire cohort of 66 cases, and was found to be 100%, 94%, 80% and 100% respectively. Inclusion of only those cases who passed the flipping view analysis improved the specificity and positive predictive value to 98% and 94%, respectively, with no change in the sensitivity and negative predictive value. Three of the four false positive overlays (i.e. graded as misaligned where true misalignment was not present in a normal eye) were classified as faint displacement and one as moderate. All 16 control cases were graded as having obvious (10/16) or moderate (6/16) true misalignment (true positives). These true and false positive groups were significantly different in the subjective grading of the obviousness of misalignment (*p* = 0.001). Regarding the magnitude of retinal displacement, three of the false positive cases were graded as having displacement of less than one vessel diameter, and one was found with a displacement greater than one vessel diameter. The magnitude of the displacement of the 16 positive control cases was significantly greater than the 4 false positive study cases (*p* = 0.01).

No significant correlation was found on univariable analysis between different variable including age, axial length measurement, the interval between imaging acquisition, the number of landmarks, laterality and gender to the primary outcome.

## Discussion

To the best of our knowledge, this is the first large validation study to assess the degree of retinal displacement in normal eyes using the homography technique. We found excellent specificity and sensitivity for the diagnosis of retinal displacement, with a significant improvement in specificity with the addition of the flipping view technique prior to the overlay analysis. This study demonstrated that two normal IR images can be accurately aligned in most cases using the overlay technique. This technique represents a promising approach for evaluating retinal displacement across various retinal pathologies. Its application extends beyond RRD cases to include other vitreoretinal surgeries such as ERM peeling and macular hole repair. Furthermore, it holds potential for assessing retinal alignment in exudative retinal pathologies such as central serous chorioretinopathy, Vogt-Koyanagi-Harada syndrome, age-related macular degeneration among others. One limitation to the widespread adoption of the overlay technique in routine clinical practice for RRD cases is the requirement for the availability of imaging data prior to RRD occurrence. However, many other retinal pathologies are commonly investigated with baseline and/or preoperative imaging studies, making it possible to study the presence and magnitude of retinal displacement.

**Fig. 2:**
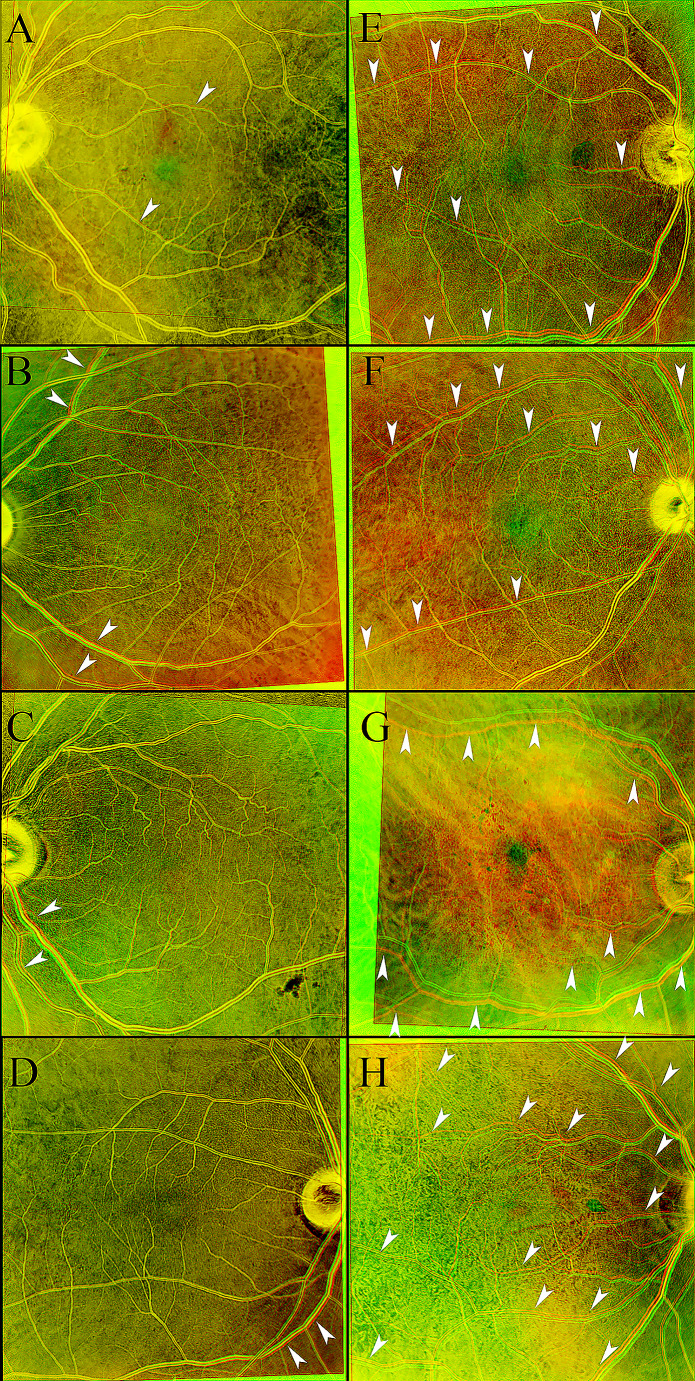
Examples of false positive cases compared to true positives of retinal displacement. There were four false positive overlays: (**a**) A single false positive overlay despite stationary and aligned ONH and choroidal vessels in the first validation step (flipping view). (**b**-**d**) Three false positive overlays with ONH and choroidal misalignment in the validation step (flipping view analysis) suggesting flaws during the manual markings. (**e**-**h**). Comparison with four true positive controls. Notice the sectoral and inconsistent apparent displacement of the vessels in the false positives compared to the consistent and overall congruent displacement in the true positive controls

It is important to find ways to minimize false positive results with the homography technique. Comparison of false and true positive cases demonstrated several differences (Fig. 2). A post hoc analysis suggested that false positives seemed to have segmental misalignment that did not follow the entire length of the blood vessel. However, true positive cases had increased displacement with increasing distance from the optic nerve which likely served as a major center of rotation [14]. We would consider excluding segmental non-angular displacement from future studies assessing retinal displacement post RRD repair using the homography technique.

Overlays with an adequate flipping view, demonstrating aligned ONH and choroidal vessels had exhibited a higher alignment rate than overlays with an unsatisfactory flipping view. We believe this is a critical and integral step into the homography technique and we advise against employing the overlay technique without incorporating the flipping view step as observed in a previous study [15]. The flipping view not only confirms adequate alignment of the ONH and deeper fixed landmarks but can also reveal sectoral misalignment that can be corrected by re-marking that sector (video on supplementary material). There was a single false positive case that passed the flipping view analysis. A review of this case revealed minor misalignment of choroidal landmarks on flipping view. This was considered not significant by the two independent graders. To avoid such false positive cases in further studies, we recommend strict criteria for flipping view analysis without even minor movements. This may further improve specificity and minimize false positive cases.

Even in cases (*n* = 11) where there were errors in the flipping view, 8 of 11 overlays were found to have retinal vessel alignment by the graders. Review of these eight cases with higher magnification revealed very small and segmental retinal vessels misalignments of less than half a vessel diameter on the overlays. This was probably missed by the graders because of the low resolution and magnification of the overlays. In other words, although flipping view demonstrated choroidal misalignment, retinal blood vessels misalignment was not obvious in the overlay. Therefore, it appears that the overlay is not as sensitive as flipping view to detect such very small segmental misalignments. Other techniques such as adaptive optics may be helpful in studying extremely small retinal misalignments [16]. Based on these results, we recommend setting a minimum threshold of half a vessel diameter for detecting retinal displacement on homography overlays. In other words, only cases that have more than half a vessel diameter of misalignment will be considered true positives. This study demonstrated that inclusion of this criterion together with the exclusion of segmental misalignment resulted in perfect specificity with no false positive cases.

Homography to overlay two retinal IR images is a new and promising technique to assess retinal displacement. Retinal displacement after RRD repair also known as low integrity retinal reattachment (LIRA) is a common and unwanted occurrence after RRD repair [8, 17, 18]. FAF is considered the gold standard for diagnosis retinal displacement and previous studies have evaluated LIRA on this basis [5]. Our group has previously utilized the homography technique to demonstrate that fundus autofluorescence (FAF) has low sensitivity (46%) (high false negative rate) for the diagnosis of postoperative retinal displacement [14]. Two previous studies found a quantitative correlation between LIRA and visual distortion [11, 17] which suggests that the higher the magnitude of LIRA the worse the visual distortion. Since the resolution of retinal displacement by the homography technique is superior to current imaging capabilities with FAF [14], it may shed new light on postoperative retinal displacement. In addition, it may help us to minimize retinal displacement by being able to better assess outcomes with different RRD repair techniques or different intraoperative techniques [19]. Minimizing LIRA may also reduce the occurrence of post-RD repair metamorphopsia and aniseikonia.

Previous studies have used different techniques to measure retinal displacement in eyes with epiretinal membrane (ERM) or macular hole [15, 20–22]. Several previous studies have used anatomic landmarks that could move together with the retina as reference points, such as retinal vessel [20–22]. Tung et al. utilized a software program for comparing the preoperative and postoperative position of the fovea and used choroidal vessels as reference points in patients undergoing surgery for epiretinal membrane [15]. Unfortunately, that study did not incorporate any method to validate the technique.

This study has some limitations. First, manual marking of the deeper layers, especially if not done meticulously, may cause errors. In case of two landmarks that do not match, the overlay will show false retinal vessel misalignment in that sector resulting in a false positive overlay. Algorithms using artificial intelligence could potentially identify choroidal and RPE landmarks making this method automatic, faster and more precise. Second, the grading assessment of both steps is subjective with no strict criteria for stationary landmarks on flipping view or evidence of retinal blood vessel misalignment on the overlay analysis. This was reflected in the suboptimal agreement rate of 88% (Cohen kappa 0.64) in the first grading step of the flipping view. However, the agreement rate in the second step was very high (98%). Another potential limitation is the extended interval between the two IR images which could span up to 227 weeks. However, the necessity of utilizing the overlay technique in patients following RRD repair often entails imaging prior to the occurrence of RRD, which is typically unavailable. In cases where such imaging is accessible, it may originate from years before the RRD event [14]. Finally, this study did not assess repeatability (intra-grader consistency) and reproducibility (inter-grader consistency) using the same imaging sets and further study is needed.

In conclusion, this study analyzed a novel homography technique on IR images and demonstrated that it has an excellent ability to properly align retinal vessels in normal eyes and has excellent sensitivity and specificity of detecting true retinal displacement. A better understanding of retinal misalignment may allow the development of medical and surgical techniques to improve functional outcomes in the treatment of retinal diseases that cause neurosensory retinal detachment.

## Electronic supplementary material

Below is the link to the electronic supplementary material.

Supplementary Material 1

## Data Availability

Dr. Brosh, MD and all the authors had full access to all the patient data in the study and take responsibility for the integrity of the data and the accuracy of the data analysis.
